# Different doses of methimazole treatment of children and adolescents with graves’ disease: a clinical study based on 161 cases of outpatients

**DOI:** 10.1186/s12902-023-01484-2

**Published:** 2023-10-23

**Authors:** Peng Li, Wei Wang, Meiqin Yan, Xianhui Zhang, Jie Pan, Lina Gong

**Affiliations:** 1 Department of Laboratory Medicine, Shanxi Children’s Hospital, Shanxi Maternal and Child Health Hospital, Taiyuan, China; 2 Department of Laboratory Medicine, Shanxi Provincial People’s Hospital, Taiyuan, China; 3 Department of Pathology, Stanford University School of Medicine, Palo Alto, CA 94305 USA; 4 Department of Medical Risk Management, The Third Medical Center of Chinese PLA General Hospital, Beijing, 100039 China

**Keywords:** Children, Adolescents, Graves’ Disease, Methimazole, Adverse effects

## Abstract

**Objective:**

This study aimed to evaluate the association between the initial dose of MMI and the clinical course, as well as adverse effects on young people with GD.

**Methods:**

One hundred and sixty-one children and adolescents with newly diagnosed GD were enrolled for this study and categorized into four groups based on initial serum-free T3 and T4 levels and daily MMI doses: Group A (mild, 0.3–0.5 mg/kg/day, n = 78), Group B (moderate, 0.6–0.8 mg/kg/day, n = 37), Group C (severe, 0.6–0.8 mg/kg/day, n = 24), and Group D (severe, 0.8-1.0 mg/kg/day, n = 22). The thyroid function, blood cell analysis and liver function were examined before treatment and at 4, 8 and 12 weeks after treatment. Outcome of long-term follow-up were also observed.

**Results:**

After 12 weeks of treatment, 91.0% of the patients in group A and 90.9% of the patients in group D recovered to normalization of FT3, which was slightly higher than the other two groups; 70.8% of the patients in group C recovered to normalization of FT4, which was slightly lower than that in the other three groups. The incidence of minor adverse effects was 12.8% in group A, 13.5% in group B, 16.7% in group C and 40.9% in group D (P < 0.01). Remission was achieved in 38 patients (23.6%).

**Conclusions:**

Lower doses of MMI (0.3–0.5 mg/kg/day) are suitable for mild GD, and higher doses of MMI (0.6–0.8 mg/kg/day) are advisable for moderate or severe GD. Much higher doses of MMI (0.8-1.0 mg/kg/day) are harmful for initial use in children and adolescents with GD patients.

## Introduction

Graves’ disease (GD) is the most common cause of hyperthyroidism in children and adolescents, which can occur at any stage of childhood [1, 2]. The incidence of GD in children and adolescents appears to be increasing worldwide, ranging from 1.5 to 6.5 cases per 100 000 person-year [3–5]. There are currently three options for treating GD: antithyroid drugs (ATDs), surgery and radioactive iodine [2, 6–8].

The optimal treatment of GD in children and adolescents remains controversial. More clinical studies are needed to compare the frequency of failures and the short- and long-term side effects of various treatments. At present, ATD is still the first choice for children and adolescents with GD, although many of them eventually experience a relapse of GD [9]. Meanwhile, the definitive treatment (surgery or radiation) often results in permanent hypothyroidism, requiring lifelong replacement of levothyroxine [10].On the surface, the situation looks rather bleak. For clinicians, how to scientifically manage GD adolescents and counsel them about the disease course to obtain more significant benefits in the treatment process is an urgent clinical problem [11].

ATD is usually the first-line therapy for most young GD people. How to control hyperthyroidism effectively and reduce adverse reactions has always been the focus of clinical medical research. To date, the correlation between the initial dose of methimazole (MMI) and the clinical course of GD remains controversial. In particular, there are few clinical studies on MMI treating Chinese youth with GD. Therefore, in this study, different doses of MMI were selected for the treatment of young Chinese people with GD according to the severity of the disease. The short-term efficacy and related adverse effects were analyzed to provide a clinical basis for the scientific management of GD.

## Materials and methods

### Study subjects

From June 2017 to May 2022, a total of 161 children and adolescents (aged 4–15 with a mean of 9.6 ± 2.6 years) with newly diagnosed GD were enrolled in the study at the Children’s Hospital of Shanxi Province. The subjects were divided into three grades based on the initial serum-free T4 and T3 (FT3 and FT4) concentrations. The levels of FT3 and FT4 were three times lower than the upper limit of the standard reference value in mild patients, four times higher than the upper limit in severe patients, and between mild and severe patients in moderate patients.

### Detection methods

The hormone concentrations including FT3 (normal range 5.1–7.4 pmol/L, reportable range 0.3–30.8 pmol/L) and FT4 (normal range 11.1–18.1 pmol/L, reportable range 1.3–155 pmol/L) were measured by chemiluminescence assay on Siemens ADVIA Centaur XP automatic electrochemiluminescence analyzer. Thyrotrophin receptor antibody (normal range < 1.75IU/L, reportable range 0.3–40 IU/L) were measured by chemiluminescence assay on Roche COBAS e601 automatic electrochemiluminescence analyzer. The alanine aminotransferase (ALT), aspartate aminotransferase (AST) and total bilirubin (TBIL) were determined by the enzymic method on Beckman Coulter AU5800 automatic biochemical analyzer. Leukocyte differential counts were performed by standard procedures on a Sysmex XN-350 automated hematology analyzer.

### Methimazole Treatment and Monitoring

In this research, the subjects were divided into four groups according to the initial serum-free T3 and T4 concentrations and daily doses of MMI. Group A included mild patients treated with MMI according to the daily dose shown in the guideline (mild group, lower dose, 0.3–0.5 mg/kg/day, n = 78) [12]. Group B consisted of moderate patients who received MMI at higher doses (moderate group, higher dose, 0.6–0.8 mg/kg/day, n = 37). Group C consisted of severe patients treated with MMI at higher doses (severe group, higher dose, 0.6–0.8 mg/kg/day, n = 24). Group D consisted of severe patients receiving MMI at much higher doses (severe group, much higher dose, 0.8-1.0 mg/kg/day, n = 22).

When thyroid hormone levels are normal, MMI doses can be reduced to a lower dose (0.3–0.5 mg/kg/day) to maintain a euthyroid state. The thyroid function, blood cell analysis and liver function were detected before treatment and at 4, 8 and 12 weeks after treatment. Remission was defined as maintenance a normal thyroid function for more than 12 months after discontinuation of methimazole treatment [13].

### Statistical analyses

Continuous variables were described as medians and interquartile ranges, and categorical variables were presented as counts and percentages. As appropriate, data were analyzed statistically using chi-squared analysis, Kruskal–Wallis H-test and Mann–Whitney U-test. all data were analyzed using the SPSS software version 22.0 (IBM Company, Chicago, IL, United States), and the two-sided p-value less than 0.05 was considered statistically significant.

## Results

### General Information and Clinical Feature

**Fig. 1:**
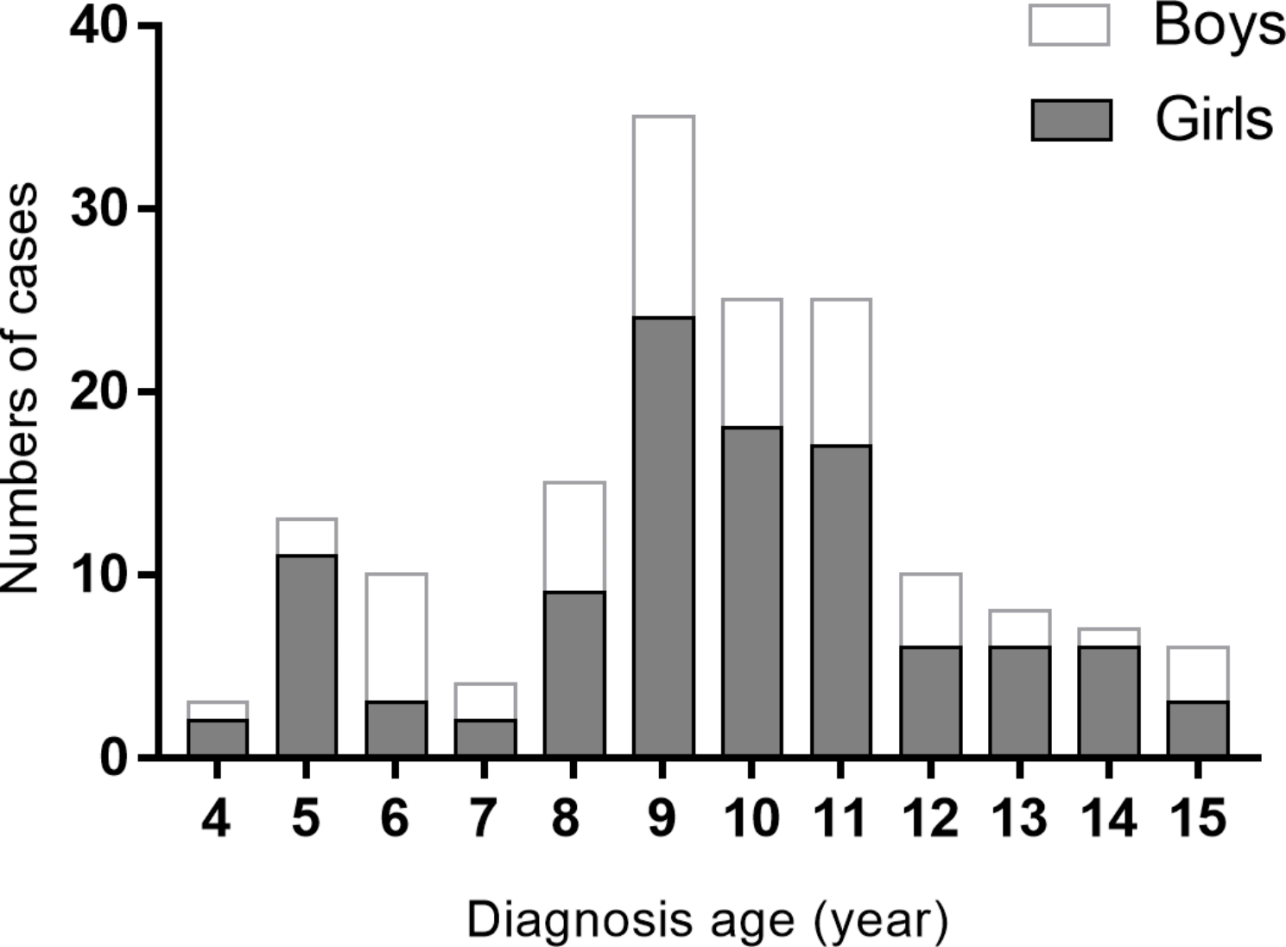
Distribution of 161 children and adolescents with GD according to age and sex at diagnosis

The mean age of patients at initial diagnosis was 9.6 ± 2.6 years (aged 4–15). There were 54 boys (33.5%) and 107 girls (66.5%), with a male-to-female ratio of 1/1.98. GD in children can occur at any age, with a high incidence in adolescence (Fig. 1). The mean age, gender ratio and serum FT3 and FT4 concentrations at the time of diagnosis of GD are shown in Table 1. Serum FT3 level in group A was significantly lower than in group B (*P* < 0.01). The level of serum FT4 in group A was significantly lower than that in other groups (*P* < 0.01), and the FT4 level in group B was significantly lower than that in group C (*P* < 0.01) and group D (*P* < 0.01). There was no significant difference in serum FT4 levels between group C and group D (*P* = 0.414).

**Table 1:** Clinical features and laboratory findings at initial diagnosis in children and adolescents with GD

	n	Female /male	Age (years)	FT3 (pmol/L)	FT4 (pmol/L)	TRAb (IU/L) ^b^
Group A (mild group, lower dose)	78	50/28	10.0 ± 2.2	9.6(7.7–11.9)	26(20.0-33.3)	15.84(9.39–22.39)
Group B (moderate group, higher dose)	37	27/10	9.4 ± 2.3	25.2(22.5–30.0)	56.5(52.4–60.7)	30.31(23.38–34.64)
Group C (severe group, higher dose)	24	15/9	8.4 ± 3.5	>30.8 ^a^	96.1(84.1-137.5)	36.27 (26.47-40.0) ^c^
Group D (severe group, much higher dose)	22	15/7	9.4 ± 3.2	>30.8 ^a^	118.3(83.5-154.2)	37.25 (25.31-40.0) ^c^
*P* ^*d*^		0.78	0.08	<0.01	<0.01	<0.01

^a^ Reportable range of FT3 was 0.3–30.8 pmol/L

^b^ TRAb: thyrotrophin receptor antibody

^c^ Reportable range of TRAb was 0.3–40 IU/L

^d^ FT3 and TRAb of group C and group D were not counted

### Serum FT3 and FT4 in different groups after treatment (MMI efficacy)

We analyzed the level of serum FT3 and FT4 in different groups after treatment (Tables 2 and 3). After four weeks of treatment, 78.2% of patients recovered to normalization of FT3, and 74.4% recovered to normalization of FT4 in group A, which was significantly higher than that in the other three groups. After eight weeks of treatment, 89.7% of patients in group A and 86.4% of patients in group D recovered to normalization of FT3, which was slightly higher than the other two groups; 78.2% of patients in group A and 81.8% of patients in group D recovered to normalization of FT4, which was slightly higher than the other two groups. After 12 weeks of treatment, 91.0% of the patients in group A and 90.9% in group D recovered to normalization of FT3, which was slightly higher than those in the other two groups; 70.8% of the patients in group C returned to normalization of FT4, which was marginally lower than that in the other three groups.

**Table 2:** Serum FT3 at different times after treatment

	n		4w		8w		12w	
		FT3 (pmol/L)		Normalization [(n(%)]	FT3 (pmol/L)	Normalization [(n(%)]	FT3 (pmol/L)	Normalization [(n(%)]
Group A (mild group, lower dose)	78		6.2(5.2–7.1)	61(78.2%)	5.9(5.3–6.7)	70(89.7%)	5.9(5.3–6.6)	71(91.0%)
Group B (moderate group, higher dose)	37		8.9(5.7–13.2)	16(43.2%)	6.5(5.4–7.7)	28(75.7%)	5.6(4.9–6.9)	29(78.4%)
Group C (severe group, higher dose)	24		10.0(7.4–17.4)	6(25.0%)	6.2(4.9–6.9)	18(75.0%)	6.1(5.6–6.9)	19(79.2%)
Group D (severe group, much higher dose)	22		8.1(6.6–12.1)	8(36.4%)	6.3(5.8–6.8)	19(86.4%)	5.9(5.1–6.6)	20(90.9%)
*P*			<0.01	<0.01	0.24	0.15	0.72	0.18

**Table 3:** Serum FT4 at different times after treatment

	n	4w		8w		12w	
		FT4 (pmol/L)	Normalization [(n(%)]	FT4 (pmol/L)	Normalization [(n(%)]	FT4 (pmol/L)	Normalization [(n(%)]
Group A (mild group, lower dose)	78	15(12.7–18.7)	58(74.4%)	14.5(12.8–17.6)	61(78.2%)	15.7(13.5–17.6)	63(80.8%)
Group B (moderate group, higher dose)	37	22.3(13.1–34.8)	16(43.2%)	15.9(11.1–18.3)	27(73.0%)	16.3(14.1–18.0)	29(78.4%)
Group C (severe group, higher dose)	24	23.1(17.2–35.8)	10(41.7%)	14.9(11.8–18.4)	17(70.8%)	16.4(13.4–19.2)	17(70.8%)
Group D (severe group, much higher dose)	22	17.2(14.0-31.8)	12(54.5%)	15.7(12.3–17.3)	18(81.8%)	15.9(12.4–17.8)	18(81.8%)
*P*		<0.01	<0.01	0.99	0.76	0.59	0.75

### Adverse effects after the start of MMI

28 of 161 (17.4%) patients experienced minor adverse effects (such as rash, liver dysfunction, transient neutropenia, arthralgia and myalgia), and no serious adverse effects (such as aplastic anemia, agranulocytosis or severe hepatitis) were observed.

The incidence of minor adverse effects was 12.8% in group A, including thirteen episodes (two skin rash, six liver dysfunction, three transient neutropenia and two arthralgia/myalgia) in ten patients. In group B, the incidence was 13.5%, including five episodes (three liver dysfunction, one transient neutropenia and one arthralgia/myalgia) in five patients. The incidence was 16.7% in group C, including six episodes (three skin rash, two liver dysfunction and one transient neutropenia) in four patients. In group D, the incidence was 40.9%, including twelve episodes (two skin rash, five liver dysfunction, three transient neutropenia and two arthralgia/myalgia) in nine patients. The incidence of minor adverse effects in group D was significantly higher than in the other three groups (Table 4).

**Table 4:** Adverse effects after the start of MMI

	n	Skin rash	Liver dysfunction	Transient neutropenia	Arthralgia/myalgia	Patients with adverse effects	*P* ^a^	
Group A (mild group, lower dose)	78	2 (2.56%)	6 (7.69%)	3 (3.85%)	2 (2.56%)	10 (12.82%)	*P* < 0.01	
Group B (moderate group, higher dose)	37	0	3 (8.11%)	1 (2.70%)	1 (2.70%)	11 (13.51%)	*P* < 0.01	
Group C (severe group, higher dose)	24	3 (12.50%)	2 (8.33%)	1 (4.17%)	0	12 (16.67%)	*P* < 0.01	
Group D (severe group, much higher dose)	22	2 (9.09%)	5 (22.73%)	3 (13.64%)	2 (9.09%)	13 (40.91%)	Referent	

^a^ Comparisons of the incidence rates of adverse effects in each group with Group D

### Outcome of long-term follow-up

In 161 patients, 80 patients (49.7%) were still on medication, and 43 patients (26.7%) were lost to follow-up by the end of the observation. Remission (maintenance of euthyroidism for more than 12 months after discontinuing methimazole treatment) were achieved in 38 patients (23.6%) [20 (25.6%) in group A, 10 (27.0%) in group B, 4 (16.7%) in group C, and 4 (18.2%) in group D)]. Long-term remission was achieved in 57.9% (n = 22) of these 38 patients for more than 2 years: 13 in group A, 6 in group B, 1 in group C, and 2 in group D.

## Discussion

Most pediatric endocrinologists recommend MMI as initial treatment in the hope that the patient’s Graves’ disease will be resolved [1, 8, 11]. The typically used MMI dose is 0.2 to 0.5 mg/kg/day. For severe clinical or biochemical hyperthyroidism, doses exceeding 50–100% may be employed. The fundamental principle followed with MMI is to begin with a high dosage, then gradually reduce and maintain it [12]. While the initial dosage of MMI may vary based on the severity of Graves’ disease, there is a scarcity of clinical research investigating the effectiveness and potential adverse events associated with these initial dosages concerning disease severity, especially in the context of Chinese children and adolescents. In our clinical practice, we have also observed an increased occurrence of adverse effects in children and adolescents with severe hyperthyroidism who are given high doses of MMI as initial treatment [14, 15]. Therefore, our study selected varying MMI doses for the treatment of young Chinese individuals with Graves’ disease, taking into account disease severity. The study aimed to analyze short-term efficacy and associated adverse effects, offering a clinical foundation for the scientific management of Graves’ disease.

The initial dosage of MMI is determined based on the severity of GD, whereas majority of previous research in this area has largely focused on adults. Nakamura et al. [16] categorized GD patients into mild and severe cases based on their pre-treatment blood FT4 levels, administering daily doses of MMI 30 mg and 15 mg, respectively. During a 12-week observation period, it was found that MMI 15 mg/day was suitable for mild GD cases, with comparable efficacy to 30 mg/day but with a lower incidence of adverse reactions. MMI 30 mg/day, on the other hand, was recommended for severe GD cases. Shotaro Sato et al. [17]found that for patients with moderate to severe hyperthyroidism, both a daily regimen of 15 mg of MMI combined with 38 mg of inorganic iodine and a daily regimen of 30 mg of MMI as initial treatment were effective. Nevertheless, the use of 15 mg of MMI combined with 38 mg of inorganic iodine daily may offer superior liver protection. Hyun Gyung Lee et al. [14] conducted a retrospective investigation into the clinical progression of children and adolescents with GD. Their study elucidated that lower MMI dosages are appropriate for the management of mild cases of Graves’ disease. The administration of high MMI dosages (> 0.7 mg/kg/day) as an initial treatment option for children and adolescents with Graves’ disease should be carefully reconsidered.

In this study, we devised a more refined grouping approach to assess the short-term efficacy of different MMI dosages in treating children and adolescents with GD of varying severity. Participants were categorized into four distinct groups based on their initial serum-free T3 and T4 concentrations, as well as their daily MMI doses. Group A included mild patients, Group B consisted of moderate patients, and groups C and D were severe. Patients in group A received lower doses of MMI (0.3–0.5 mg/kg/day), those in groups B and C received a higher dose of MMI (0.6–0.8 mg/kg/day), and those in group D were treated with a much higher dose of MMI (0.8-1.0 mg/kg/day). In terms of the results, first of all, the rate of thyroid hormone levels in group A recovered to normalization was high, indicating that a lower dose of MMI was sufficient for patients with mild hyperthyroidism. Second, the rate of hormone returned to normal in group C was lower than that in group B four weeks after treatment, but there was no significant difference at other time points. These results indicated that with higher doses of the MMI, the initial duration that induced a euthyroid state was longer in severe patients than in moderate patients, but there was no difference over time. The third, severe patients treated with a much higher dose of MMI (group D), showed a slightly higher rate that induced a euthyroid state than severe patients treated with a higher dose of MMI (group C) at different time points, but there was no statistically significant difference (*P*>0.05). In conclusion, as long as the dose of MMI is given according to the thyroid hormone levels before treatment, the same short-term efficacy can be achieved. However, a much higher doses (0.8-1.0 mg/kg/day) did not shorten the time required to normalize hormone in severe patients compared to higher doses (0.6–0.8 mg/kg/day).

MMI-related adverse effects include rash, liver dysfunction, transient neutropenia, arthralgia, myalgia, aplastic anemia, agranulocytosis and severe hepatitis, etc. Several reports suggest that MMI-induced adverse effects depend on the dosage of MMI administered, these results are similar to our findings in children [8, 11, 18, 19]. No serious adverse effects were observed in this study. The incidences of minor adverse effects were 12.8%, 13.5% and 16.7% in groups A, B, and C, respectively. There were no differences among these three groups. However, the incidence rate was 40.9% in group D, which was significantly higher than in the other three groups (P<0.05). When we compared the severe patients treated with a higher dose of MMI (group C) and with a much higher dose of MMI (group D), we found no significant difference in the remission rate between the 2 groups (P>0.05). Therefore, a much higher dose of MMI (0.8-1.0 mg/kg/day) should be avoided in the initial treatment of young people with GD.

Remission for more than 12 months was achieved in 38 patients (23.6%) [20 (25.6%) in group A, 10 (27.0%) in group B, 4 (16.7%) in group C, and 4 (18.2%) in group D)]. The remission rates in the group A and B were higher may because these group included many cases of mild and moderate GD [15]. Long-term remission was achieved in 57.9% (n = 22) of these 38 patients for more than 2 years: 13 in group A, 6 in group B, 1 in group C, and 2 in group D. The long-term outcomes did not differ between the higher dose group (group C) and much higher dose group (group D). In conclusion, remission rate with ATD treatment in children and adolescents was not high, consistent with previous reports [10, 13].

Limited by this study’s short-term design, it is unsuitable for observing the long-term failure frequency and side effects of MMI treatment. Further long-term clinical observation is needed. In summary, our data show that lower doses of MMI (0.3–0.5 mg/kg/day) are appropriate for mild GD and higher doses of MMI are suitable for moderate or severe GD (0.6–0.8 mg/kg/day). Initial use of much higher doses of MMI (0.8-1.0 mg/kg/day) is harmful in children and adolescents with GD.

## Data Availability

The datasets generated during the study are available from the corresponding author on reasonable request.
